# Myeloid neoplasm with eosinophilia associated with *FIP1L1–PDGFRA* presenting as femoral avascular necrosis: a rare case report

**DOI:** 10.1016/j.htct.2026.106258

**Published:** 2026-02-08

**Authors:** Katia Gleicielly Frigotto, Ingrid Caroline Rosa Diogo, Pedro Guilherme Mol da Fonseca, Victor Lopes de Abreu, Júlia Rosa Dantas, Paula Santos Barroso, Juliana Bastos Torres, Natália Laso Fonseca, Vitor Ribeiro Gomes de Almeida Valviesse

**Affiliations:** a Universidade Federal do Estado do Rio de Janeiro (UNIRIO), Hospital Universitário Gaffrée e Guinle, R. Mariz e Barros, 775 - Maracanã, Rio de Janeiro RJ, 20270-004, Brazil; b Instituto Nacional de Câncer José Alencar Gomes da Silva (INCA), Pr. da Cruz Vermelha, 23 - Centro, Rio de Janeiro RJ, 20230-130, Brazil

## Introduction

Myeloid neoplasms with eosinophilia and abnormalities of the *platelet-derived growth factor receptor alpha (PDGFRA)* gene are rare hematologic malignancies driven by fusion genes that encode constitutively active tyrosine kinases [1]. Although they most commonly present as chronic eosinophilic leukemia, these disorders may also manifest as acute myeloid leukemia or T-lymphoblastic leukemia/lymphoma [1].

Clinically, these disorders are characterized by multisystem involvement resulting from the infiltration of tissues by clonal eosinophils. The heart, lungs, skin, gastrointestinal tract, and central or peripheral nervous system are among the most frequently affected organs 2, 3, 4, 5, 6. The *Factor Interacting with PAPOLA and CPSF1* (*FIP1L1*)*-PDGFRA* fusion gene, resulting from a cryptic microdeletion on chromosome 4q12, plays a central role in disease pathogenesis and predicts a remarkable therapeutic response to imatinib, which has significantly improved outcomes for affected patients [7].

Despite the broad range of organ involvement described, skeletal manifestations remain exceptionally uncommon [8,9]. Only isolated case reports have documented bone infiltration or lytic lesions associated with *FIP1L1-PDGFRA*-positive neoplasms, underscoring the rarity of this presentation [8,9]. Notably, avascular necrosis has not been previously reported as an initial manifestation of this disease.

We describe a rare case of a *FIP1L1-PDGFRA*-rearranged myeloid neoplasm presenting initially with femoral avascular necrosis, highlighting the diagnostic challenges, imaging findings, and response to targeted therapy.

## Case presentation

A 59-year-old man with no relevant medical, family, social, or occupational history presented with progressive asthenia and severe right hip and lower-limb pain lasting five months. The pain gradually worsened, compromising ambulation. He also reported an unintentional weight loss of approximately 20 kg during this period. He initially sought emergency care, where he required a transfusion of red blood cell concentrate, and was subsequently referred to a tertiary university hospital for further evaluation.

Physical examination revealed intense pain on mobilization of the right lower limb. Initial laboratory testing showed hemoglobin of 8.2 g/dL, leukocyte count of 67.2 × 10^9^/L with an absolute eosinophil count of 18.141 × 10^9^/L, platelet count of 107,000/mm³, and erythrocyte sedimentation rate of 111 mm/h.

**Fig. 1:**
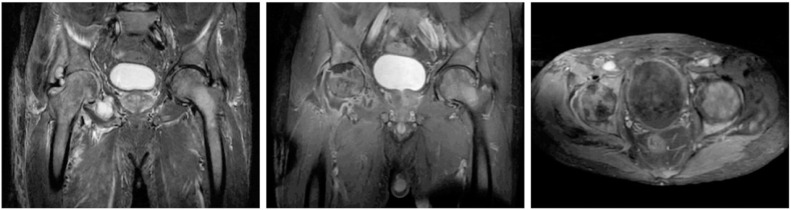
Magnetic resonance imaging of the pelvis showing avascular necrosis of the right femoral head, chondral thinning and narrowing of the right hip joint space, large right hip joint effusion with distension of the articular recesses and capsular enhancement after contrast administration; in addition to edema in the gluteal muscles and the proximal right thigh, along with signs of bone marrow infiltration of the pelvis, sacrum, studied vertebral bodies, and bilaterally of the femurs.

Abdominal computed tomography demonstrated splenomegaly (16 cm longitudinal axis). Magnetic resonance imaging of the pelvis and right knee revealed avascular necrosis of the right femoral head without loss of sphericity, associated with chondral thinning and joint space narrowing. A large right hip joint effusion was present, with distension of the articular recesses and capsular enhancement after contrast. Edema of the gluteal muscles and proximal thigh was noted, along with diffuse bone marrow infiltration of the pelvis, sacrum, evaluated vertebral bodies, and both femurs (Figure 1).

**Fig. 2:**
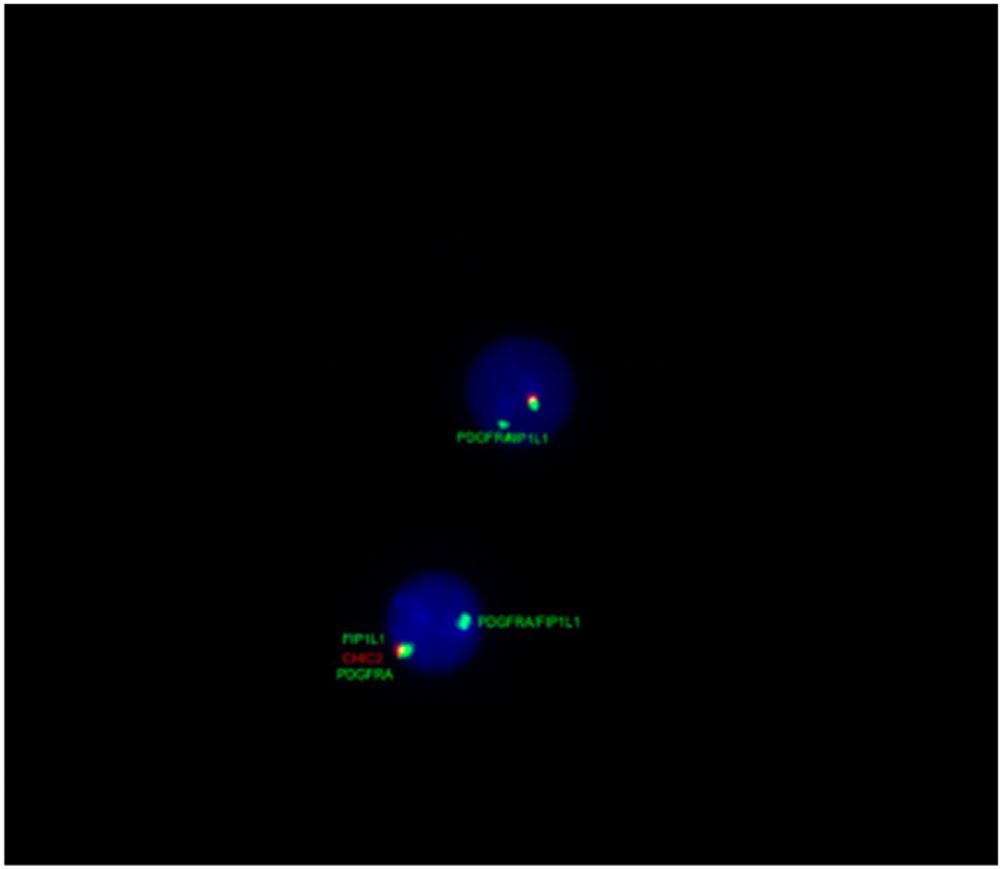
Fluorescence in situ hybridization for 4q12 (*FIP1L1/PDGFRA*) on chromosome 4 - rearrangement of *FIP1L1-PDGFRA* was detected in 98 % of the analyzed nuclei.

Bone marrow immunophenotyping showed eosinophilia (22 %) with predominance of granulocytic lineage. Tests for *BCR::ABL1* and *JAK2* were negative, while fluorescence in situ hybridization (FISH) revealed the *FIP1L1-PDGFRA* rearrangement in 98 % of analyzed nuclei (Figure 2). A biopsy of the right femur confirmed bone infiltration compatible with chronic eosinophilic leukemia.

The patient initially received hydroxyurea for cytoreduction. After five months, therapy was switched to **imatinib (400 mg/day)**, resulting in marked clinical improvement and the normalization of hematologic parameters. Complete radiologic resolution of bone lesions was achieved after six months of imatinib treatment.

## Discussion

Myeloid and lymphoid neoplasms associated with eosinophilia and the *FIP1L1-PDGFRA* rearrangement are rare hematologic malignancies that typically present with persistent eosinophilia and multisystem involvement, most commonly affecting the heart, lungs, skin, and central or peripheral nervous system [1, 2, 3,5,7]. In contrast, skeletal involvement is exceptionally uncommon, with only isolated case reports describing bone lesions secondary to these neoplasms [8,9].

Recent literature suggests that bone infiltration may occur more frequently than previously recognized, although still representing a minority of cases. Lv et al. [8] described a young adult with multiple osteolytic vertebral lesions at presentation, while McGregor et al. [9] reported a pathological fracture due to a lytic lesion of the femoral neck in a patient with the *FIP1L1-PDGFRA* rearrangement identified in the tumor tissue despite negative bone marrow findings. These reports, together with the present case, indicate that skeletal manifestations should be considered within the clinical spectrum of *FIP1L1-PDGFRA*-positive neoplasms.

The mechanisms underlying bone involvement remain unclear. It has been proposed that eosinophilic infiltration may directly contribute to bone damage, given the release of pro-inflammatory cytokines and cytotoxic mediators by neoplastic eosinophils. Additionally, cooperating genetic alterations, such as *RUNX1* loss-of-function mutations, may enhance osteoclast activity and pathological bone resorption [8]. This aligns with the established concept that osteoclasts originate from erythroid-myeloid progenitors and can be replenished by monocyte-lineage cells derived from hematopoietic stem cells, suggesting a potential for lineage plasticity among malignant cells [8].

To our knowledge, avascular necrosis has not previously been described as an initial manifestation of the *FIP1L1-PDGFRA*-rearranged disease. In this case, the avascular necrosis likely reflected extensive marrow infiltration and local tissue damage, expanding the recognized clinical spectrum of these neoplasms. This underscores the importance of considering clonal eosinophilic disorders in patients with unexplained eosinophilia and atypical bone findings.

Diagnosis requires exclusion of secondary causes of eosinophilia, followed by molecular testing for the *FIP1L1-PDGFRA* fusion gene, an alteration undetectable by standard cytogenetics and requiring techniques such as FISH or reverse transcription polymerase chain reaction (RT-PCR) [1]. In our patient, the rearrangement was detected in 98 % of nuclei by FISH, confirming the diagnosis and guiding targeted therapy.

Imatinib mesylate has dramatically transformed the prognosis of patients with *FIP1L1-PDGFRA* rearrangements. Baccarani et al. [4] demonstrated universal hematologic and molecular responses, even at low doses, and similar findings were described by Müller et al. [5] and Hilal et al. [7], including cases with significant organ involvement. Consistent with these observations, our patient exhibited marked clinical, radiologic, and hematologic improvements following treatment with imatinib, reinforcing its role as first-line treatment regardless of disease presentation or site of involvement.

This case emphasizes the necessity of considering myeloid neoplasms with eosinophilia in the differential diagnosis of unexplained avascular necrosis, especially when associated with marked eosinophilia. Early identification of the *FIP1L1-PDGFRA* rearrangement enables timely initiation of targeted therapy, which is frequently associated with favorable outcomes even in cases with atypical presentations.

## Conclusion

Myeloid neoplasms with eosinophilia and the *FIP1L1-PDGFRA* rearrangement are rare disorders that should be considered in patients presenting with eosinophilia and evidence of target-organ damage. This case highlights an atypical and previously unreported presentation characterized by femoral avascular necrosis associated with diffuse marrow infiltration. Early recognition of this unusual manifestation is essential, as timely molecular diagnosis enables initiation of targeted therapy with imatinib, which has been consistently associated with favorable clinical outcomes.

## Author contributions

KGF: Conceptualization; Data curation; Writing – original draft. ICRD: Investigation; Visualization; Data curation. PGMF: Visualization; Writing – review & editing; Validation. VLA: Writing – original draft; Writing – review & editing. JRD: Methodology; Writing – original draft; Writing – review & editing. PSB: Methodology; Writing – original draft; Writing – review & editing. JBT: Methodology; Writing – original draft; Writing – review & editing. NLF: Investigation; Validation; Supervision. VRGAV: Conceptualization; Project administration; Supervision; Writing – review & editing.

## Ethics approval statement

The study was approved by the local institutional ethics committee (CAAE: 81,586,924.4.0000.5238; number: 7.095.793).

## Data availability

The authors confirm that the data generated and analyzed in this study are included in this published article. The data that support the findings of this study are available from the corresponding author upon reasonable request.

## Funding

The authors did not receive any financial support for the purpose of this case report.

## Conflicts of interest

No conflicts of interest declared.
